# Interaction between the transmembrane domains of Sho1 and Opy2 enhances the signaling efficiency of the Hog1 MAP kinase cascade in *Saccharomyces cerevisiae*

**DOI:** 10.1371/journal.pone.0211380

**Published:** 2019-01-25

**Authors:** Tomomi Takayama, Katsuyoshi Yamamoto, Haruo Saito, Kazuo Tatebayashi

**Affiliations:** 1 Division of Molecular Cell Signaling, Institute of Medical Science, The University of Tokyo, Tokyo, Japan; 2 Laboratory of Molecular Genetics, Frontier Research Unit, Institute of Medical Science, The University of Tokyo, Tokyo, Japan; 3 Department of Biological Sciences, Graduate School of Science, The University of Tokyo, Tokyo, Japan; Kindai University, JAPAN

## Abstract

To cope with increased extracellular osmolarity, the budding yeast *Saccharomyces cerevisiae* activates the Hog1 mitogen-activated protein kinase (MAPK), which controls a variety of adaptive responses. Hog1 is activated through the high-osmolarity glycerol (HOG) pathway, which consists of a core MAPK cascade and two independent upstream branches (SHO1 and SLN1 branches) containing distinct osmosensing machineries. In the SHO1 branch, a homo-oligomer of Sho1, the four-transmembrane (TM) osmosensor, interacts with the transmembrane co-osmosensors, Hkr1 and Msb2, and the membrane anchor protein Opy2, through their TM domains, and activates the Ste20-Ste11-Pbs2-Hog1 kinase cascade. In this study, we isolated and analyzed hyperactive mutants of Sho1 and Opy2 that harbor mutations within their TM domains. Several hyperactive mutations enhanced the interaction between Sho1 and Opy2, indicating the importance of the TM-mediated interaction between Sho1 and Opy2 for facilitating effective signaling. The interaction between the TM domains of Sho1 and Opy2 will place their respective cytoplasmic binding partners Pbs2 and Ste11 in close proximity. Indeed, genetic analyses of the mutants showed that the Sho1-Opy2 interaction enhances the activation of Pbs2 by Ste11, but not Hog1 by Pbs2. Some of the hyperactive mutants had mutations at the extracellular ends of either Sho1 TM4 or Opy2 TM, and defined the Sho1-Opy2 binding site 1 (BS1). Chemical crosslinking and mutational analyses revealed that the cytoplasmic ends of Sho1 TM1 and Opy2 TM also interact with each other, defining the Sho1-Opy2 binding site 2 (BS2). A geometric consideration constrains that one Opy2 molecule must interact with two adjacent Sho1 molecules in Sho1 oligomer. These results raise a possibility that an alteration of the conformation of the Sho1-Opy2 complex might contributes to the osmotic activation of the Hog1 MAPK cascade.

## Introduction

Extreme environmental osmotic conditions are major threats to their survival for free living single-celled organisms such as the budding yeast *Saccharomyces cerevisiae*. To cope with increased external osmolarity, yeast initiates coordinated adaptive responses that include the synthesis, uptake, and intracellular retention of the compatible osmolyte glycerol [1–5], changes in the global pattern of gene expression and protein synthesis [6–8], and temporary arrest of the cell cycle at multiple phases to gain time for adaptation [9–11]. These adaptive responses are governed by the Hog1 MAP kinase (MAPK). Thus, a *hog1*Δ mutant cell is highly osmosensitive, and cannot survive even under conditions of moderately high osmolarity such as 0.4 M NaCl.

Hog1 is activated through the High Osmolarity Glycerol (HOG) signaling pathway, which is composed of upstream osmosensing mechanisms, a central signal transduction MAP kinase (MAPK) module, and downstream effector functions [12–14]. MAPK modules are evolutionarily conserved three-kinase cascades composed of a MAPK, a MAPK kinase (MAPKK), and a MAPKK kinase (MAPKKK). When activated by specific stimuli, a MAPKKK phosphorylates and thus activates a cognate MAPKK. The activated MAPKK then phosphorylates and activates a cognate MAPK [15].

**Fig 1:**
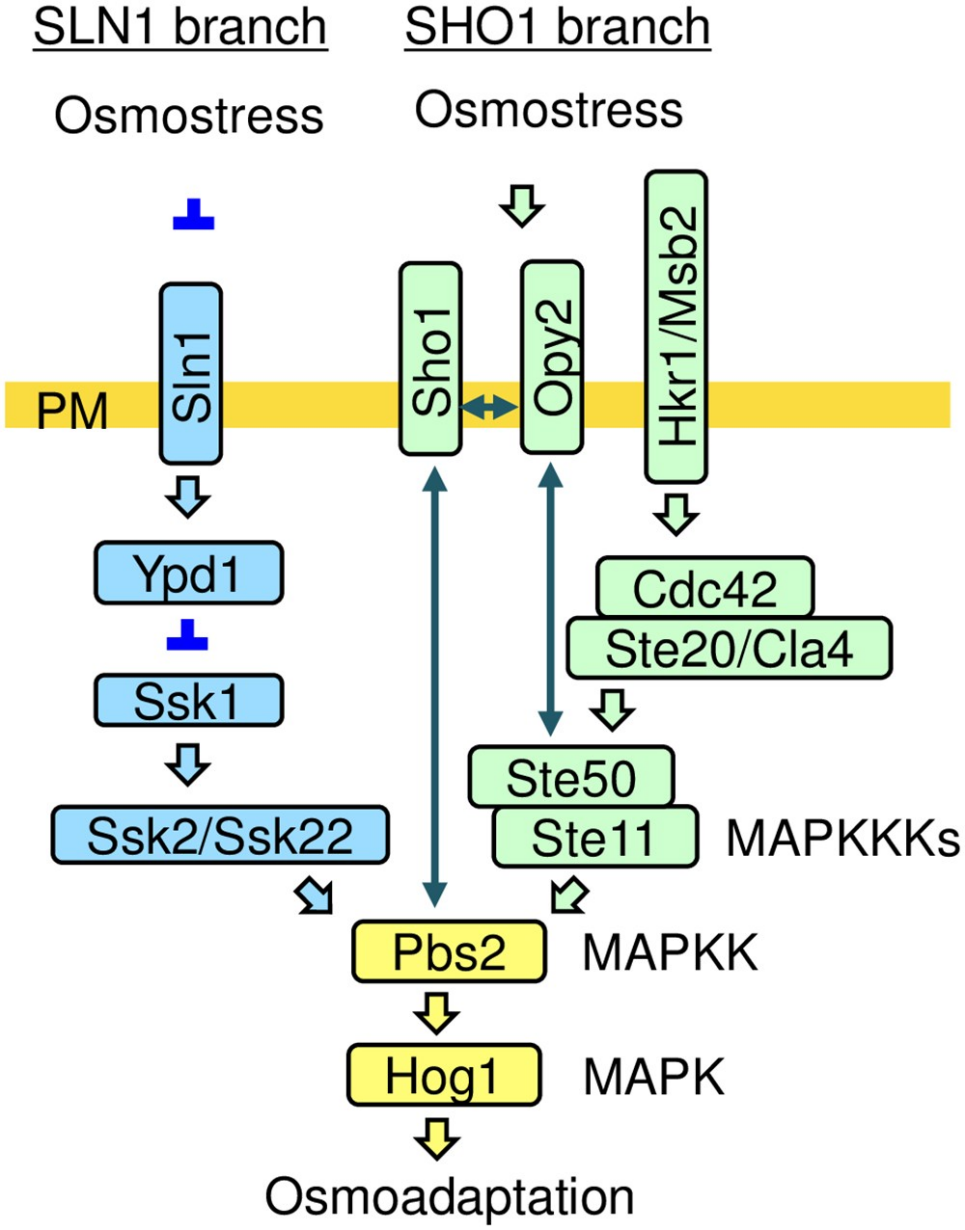
A schematic model of the HOG pathway. Proteins that are only involved in the SHO1 branch are shown in light green. Proteins that are specific to the SLN1 branch are colored light blue. Pbs2 and Hog1 are common to both the SHO1 and SLN1 branches. The proteins separated by a thrash (/) are functionally redundant. The yellow horizontal bar represents the plasma membrane (PM). Arrows indicate activation, whereas the inverted T-shaped bars represent inhibition. Double-headed arrows indicate the physical interactions of the proteins. Not all the known components or interactions are shown.

The HOG pathway employs multiple and redundant upstream osmosensing mechanisms that all lead to Hog1 activation. Specifically, upstream osmosensing signaling of the HOG pathway consists of the SLN1 branch and the SHO1 branch (Fig 1). The Ste11 MAPKKK, an upstream activator of the Hog1 MAPK in the SHO1 branch, is activated by phosphorylation by the Ste20/Cla4 kinases when osmostress is applied [16–18]. Overexpression of constitutively-active Ste11 mutants, such as Ste11-Q301P or Ste11-DDD, induces Hog1 activation even in the absence of osmostress [16, 17]. However, expressing these constitutively-active Ste11 mutant proteins at the endogenous level, *i*.*e*., by using a single-copy plasmid that carries the *STE11* promoter, does not activate Hog1 in the absence of osmostress. One possible interpretation for this observation is that osmostress is still needed to activate Hog1 even when Ste11 is activated by a non-osmotic mechanism. It is possible, for example, that osmostress somehow enhances the signaling efficiency of the Ste11-Pbs2-Hog1 MAPK cascade [17]. In the SHO1 branch of the HOG pathway, a number of non-kinase proteins (Hkr1, Msb2, Sho1, Opy2, Ahk1, Bem1, and Ste50) are involved in activation and/or regulation of the Hog1 MAPK [17, 19–24]. In this report, we describe our findings concerning the possible mechanism by which the signaling efficiency of the Ste11-Pbs2-Hog1 MAPK cascade is regulated by the interaction between the transmembrane domains of Sho1 and Opy2.

## Materials and methods

### Media and buffers

Standard yeast media and genetic procedures were previously described [25, 26]. CAD medium consists of 0.67% yeast nitrogen base (Sigma), 2% glucose, 0.5% casamino acid (Sigma) and appropriate supplements (20 μg ml^-1^ uracil and 40 μg ml^-1^ tryptophan) as needed. SC medium consists of 0.67% yeast nitrogen base and 2% glucose with an appropriate yeast synthetic drop-out medium supplement. CARaf and SRaf media are the same as CAD and SC, respectively, except that they contain 2% raffinose in place of glucose. Buffer A contains 50 mM Tris-HCl (pH 7.5), 15 mM EDTA, 15 mM EGTA, 2 mM dithiothreitol (DTT), 1 mM phenylmethylsulfonyl fluoride (PMSF), 1 mM benzamidine, 5 μg ml^-1^ leupeptin, 50 mM NaF, 25 mM β-glycerophosphate and 150 mM NaCl. Buffer C2 contains 50 mM Tris-HCl (pH 7.2), 15 mM EDTA, 15 mM EGTA, 150 mM NaCl, 1 mM PMSF, 1 mM benzamidine and 5 μg ml^-1^ leupeptin. Buffer X for crosslinking contains 50 mM Tris-HCl (pH 7.2) and 15 mM EDTA. SDS loading buffer (1x) contains 50 mM Tris-HCl (pH 6.8), 2% SDS, 0.01% Bromophenol Blue, 10% glycerol and 700 mM 2-mercaptoethanol (2-ME).

### Reagents

The following reagents were used. Cys-specific chemical crosslinker: BMH (Thermo Scientific). Detergents: Brij L23 (Sigma), Triton X-100, Tween-20 (MP Biochemical), and Digitonin (Calbiochem). Other chemicals were purchased from Sigma, Wako Pure Chemical, Nacalai Tesque, and BD.

### Antibodies

For immunoblotting, the following antibodies were used as indicated: anti-GST B-14 (Santa Cruz, sc-138) 1:1000 dilution; anti-GFP B-2 (Santa Cruz, sc-9996) 1:1000 dilution; anti-HA F-7 (Santa Cruz, sc-7392) 1:1000 dilution; anti-myc 9E10 (Santa Cruz, sc-40) 1:1000 dilution. For immunoprecipitation, anti-HA 3F10 (Roche, No. 11867431001) 4 μg ml^-1^ was used.

### Yeast strains

All yeast mutants used in this work are derivatives of the S288C strain (Table 1).

**Table 1:** Yeast strains used in this study.

Strain	Genotype	Source
FP75	*MATα ura3 leu2 trp1 his3 ssk2*::*LEU2 ssk22*::*LEU2 ste11*::*HIS3*	[17]
KT024	*MATα ura3 leu2 trp1 his3 ssk2*::*LEU2 ssk22*::*LEU2 STE11-Q301P sho1*:: *kanMX6*	This study
KT039	*MAT***a** *ura3 leu2 trp1 his3 ssk2*::*hisG ssk22*::*hisG pbs2*::*kanMX6 sho1*::*HIS3*	This study
KT265	*MATα ura3 leu2 trp1 his3 ssk2*::*LEU2 ssk22*::*LEU2 opy2*::*kanMX6 pbs2*::*natMX4*	This study
KY477	*MATα ura3 leu2 trp1 his3 ssk2*::*LEU2 ssk22*::*LEU2 opy2*::*kanMX6*	[19]
KY517	*MATα ura3 leu2 trp1 his3 ssk2*::*LEU2 ssk22*::*LEU2 opy2*::*kanMX6 STE11-Q301P*	[22]
KY590-1	*MAT***a** *ura3 leu2 trp1 his3 ssk2*::*hisG ssk22*::*hisG opy2*::*natMX4 sho1*::*hphMX4*	[24]
TM257	*MATα ura3 leu2 trp1 his3 ssk2*::*LEU2 ssk22*::*LEU2*	[27]

All strains were constructed in our laboratory, and are derived from S288C.

### Plasmid constructs

Deletion and missense mutants were constructed using PCR-based oligonucleotide mutagenesis, and were confirmed by nucleotide sequence determination.

#### Vector plasmids

pRS414, pRS416, p414GAL1, p416GAL1, p416GAL1-GST, YCpIF16, and YCplac22I’ have been described [26, 28–32].

#### Sho1 plasmids

pRS416-Sho1 (= *P*_*SHO1*_*-SHO1*, *URA3*, *CEN6*) is a full-length *SHO1* genomic DNA clone, and expresses Sho1 under the control of the *SHO1* promoter. p416GAL1-Sho1 (= *P*_*GAL1*_*-SHO1*, *URA3*, *CEN6*) expresses Sho1 under the control of the *GAL1* promoter. p416GAL1-GST-Sho1 (= *P*_*GAL1*_*-GST-SHO1*, *URA3*, *CEN6*) expresses N-terminally GST-tagged Sho1 (GST-Sho1) under the control of the *GAL1* promoter. YCpIF16-Sho1 (= *P*_*GAL1*_-*HA-SHO1*, *TRP1*, *CEN4*) encodes N-terminally HA-tagged Sho1 (HA-Sho1) under the control of the *GAL1* promoter.

#### Opy2 plasmids

pRS414-Opy2 (= *P*_*OPY2*_*-OPY2*, *TRP1*, *CEN6*) is a full-length *OPY2* genomic DNA clone, and expresses Opy2 under the control of the *OPY2* promoter. p414GAL1-Opy2 (= *P*_*GAL1*_*-OPY2*, *TRP1*, *CEN6*) expresses Opy2 under the control of the galactose-inducible *GAL1* promoter. p414GAL1-Opy2ΔSR1-GFP (= *P*_*GAL1*_*-OPY2ΔSR1-GFP*, *TRP1*, *CEN6*) encodes C-terminally GFP-tagged Opy2ΔSR1 under the control of the *GAL1* promoter. p416GAL1-Opy2(1–256) ΔSR1-myc (= *P*_*GAL1*_*-OPY2(1–256) ΔSR1-3xmyc*, *URA3*, *CEN6*) encodes C-terminally 3xmyc-tagged Opy2(1–256) ΔSR1 under the control of the *GAL1* promoter.

#### Pbs2 plasmids

YCplac22I’-PBS2 (= *P*_*PBS2*_*-PBS2*, *TRP1*, *CEN4*) is a full-length *PBS2* genomic DNA clone, and expresses Pbs2 under the control of the *PBS2* promoter.

### HOG reporter assay

Reporter assays using the HOG reporter plasmid pRS413-8xCRE-lacZ (= *8xCRE-lacZ*, *HIS3*, *CEN6*), pRS414-8xCRE-lacZ (= *8xCRE-lacZ*, *TRP1*, *CEN6*), pRS415-8xCRE-lacZ (= *8xCRE-lacZ*, *LEU2*, *CEN6*) or pRS416-8xCRE-lacZ (= *8xCRE-lacZ*, *URA3*, *CEN6*) have been described [17]. All reporter assays were carried out in triplicate (or more) using independent cultures.

### In vivo binding assay

Cells containing galactose-inducible expression constructs were exponentially grown in CARaf, and were cultured for an additional 2 hr after addition of 2% galactose. Cells were harvested, washed once with ice-cold buffer A without detergent, frozen in liquid nitrogen, and resuspended in 0.5 ml of buffer A containing the detergent indicated in the Figure legends. Cells were broken by vortexing with glass beads, and were centrifuged at 9,170x*g* for 10 min at 4 °C in a microcentrifuge, and supernatant (cell extract) was recovered. To precipitate the GST-tagged proteins, 600 μg of cell extract was incubated with 50 μl of glutathione-Sepharose beads for 2 hr at 4 °C. To immunoprecipitate the HA-tagged proteins, 600 μg of protein extract was first incubated with the high affinity anti-HA antibody 3F10 (Roche) for 2 hr at 4 °C, followed by further incubation with 50 μl of protein G beads for 1 hr at 4 °C. The beads were washed 3 times in the same buffer used for cell extract preparation, and resuspended in SDS loading buffer. Samples were either incubated at 50 °C (when Sho1 was analyzed) or boiled for 5min, and separated by SDS-PAGE. Proteins were detected by immunoblotting.

### Isolation of Sho1 hyperactive mutants

A DNA segment including the 5’-UTR and amino-terminal coding region of *SHO1* (nucleotide position of +1 ~ +542) was mutagenized by error-prone PCR in the presence of 0.1 or 0.2 mM MnCl_2_. KT024 (*ssk2*Δ *ssk22*Δ *sho1*Δ *STE11-Q301P*) cells carrying an attenuated *8xCRE-lacZ* reporter gene (pRS414-8xCRE-CYC^m^-lacZ) were co-transformed with the PCR products and linearized pRS416-PGAL1-SHO1 in which the codons 42–151 of *SHO1* had been removed by the MunI and Van91I double digestion. Gap-repaired plasmids were selected on CAD (w/o Ura and Trp) plates, replica-plated onto nitrocellulose membrane disks, and incubated on CAGal plates overnight to induce the plasmid-borne *SHO1* gene. Activation of the HOG MAPK pathway was assessed qualitatively using a colony-lift β-galactosidase filter assay [17]. Constitutively-active *SHO1* mutants that induced *lacZ* expression, which rendered the cells blue, were recovered.

### Chemical crosslinking of Sho1 and Opy2 in intact cells accompanied by co-precipitation

The Cys-specific chemical crosslinker BMH was added directly to an exponentially growing yeast culture at the concentration of 0.4 mM. After incubation at 30 °C for 5 min, cells were precipitated by centrifugation at 1,700x*g* for 3 min. To quench the crosslinking reaction, cell pellets were resuspended in 1 ml of Buffer C2 containing 50 mM DTT, and incubated on ice for 15 min. Cells were then precipitated by a brief spin in a microcentrifuge tube, frozen in liquid N_2_, and thawed and resuspended in 0.5 ml of buffer A containing 0.2% Triton X-100. Cells were broken by vortexing with glass beads, and were centrifuged at 9,170x*g* for 10 min at 4 °C in a microcentrifuge, and supernatant (cell extract) was recovered. To immunoprecipitate the HA-tagged proteins, 1000 μg of protein extract was first incubated with the anti-HA high affinity antibody 3F10 (Roche) for 2 hr at 4 °C, followed by further incubation with 50 μl of protein G beads for 1 hr at 4 °C. Beads were washed 3 times in Buffer A containing Triton X-100, resuspended in the SDS loading buffer, and incubated for 5 min at 50 °C. Precipitated proteins were separated by SDS-PAGE, and detected by immunoblotting.

## Results

### Isolation of hyperactive Opy2 mutants that have mutations in the TM domain

**Fig 2:**
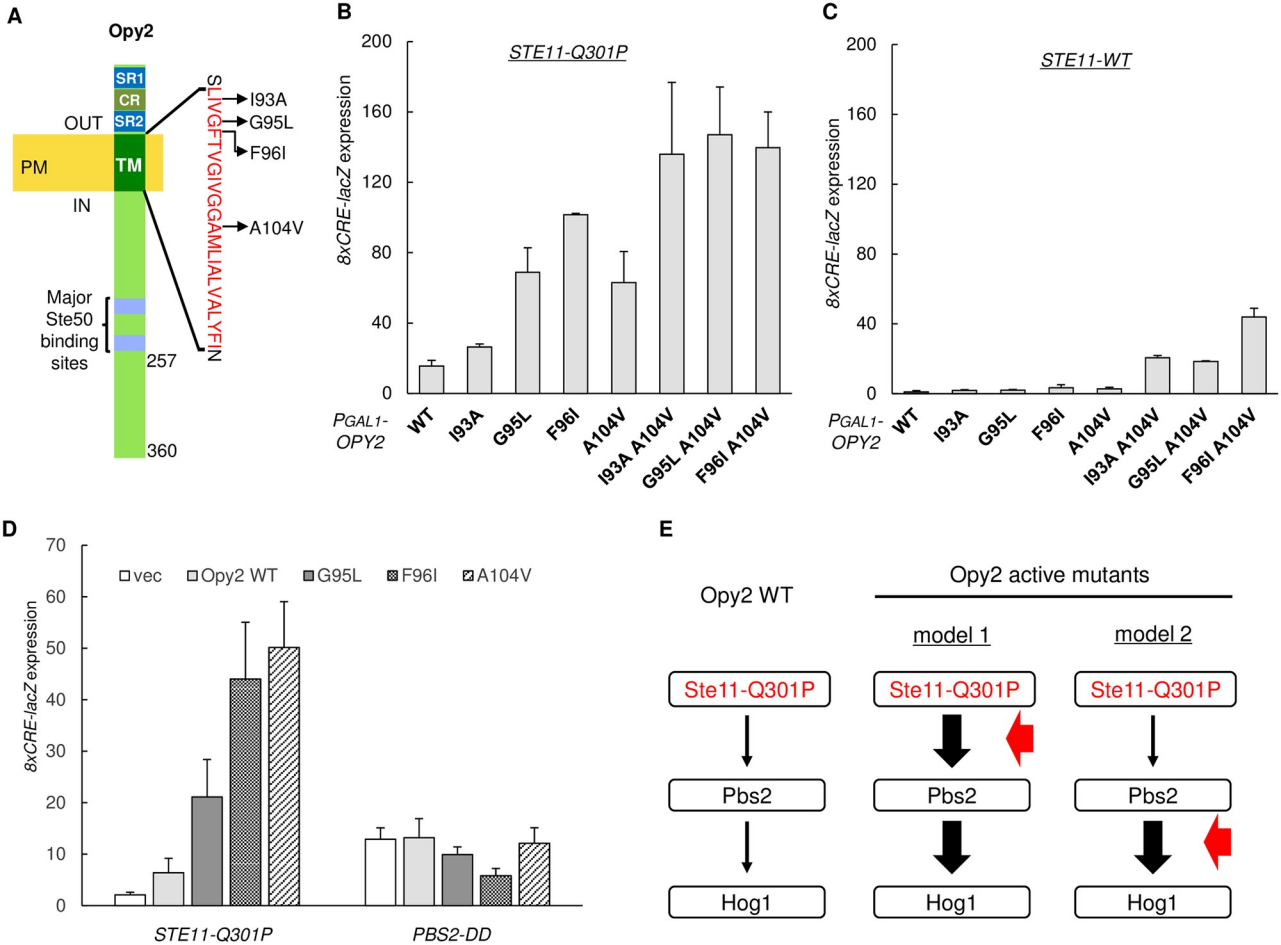
Isolation of Opy2 hyperactive mutants that have mutations in the TM domain. (**A**) Schematic structure of Opy2. Numbers indicate amino acid positions. The amino acid sequence of the TM domain is shown in red using the one-letter code. SR1 and SR2, Ser-Rich domain 1 and 2; CR, Cys-Rich domain, TM, transmembrane domain; PM, Plasma Membrane; (**B-D**) Expression of the Hog1-specific reporter gene *8xCRE-lacZ*. β-galactosidase activity is expressed in Miller units. Error bars represent S.D. (n ≥ 3). (B, C) The yeast strain KY517 (*ssk2/22*Δ *opy2*Δ *STE11-Q301P*) (B), or KY477 (*ssk2/22*Δ *opy2*Δ) (C) was transformed with a reporter plasmid carrying the *8xCRE-lacZ* gene and another plasmid that expresses the indicated Opy2 mutants from the *GAL1* promoter (P_*GAL1*_). Transformed cells were grown in CARaf, and expressions of Opy2 mutants were induced by 2% galactose for 2 hr, following which cell extracts were prepared for reporter assays to determine the expression of the *8xCRE-lacZ* gene. (D) Hog1-specific reporter assays were conducted as (B, C), except that the yeast KT265 (*ssk2/22*Δ *opy2*Δ *pbs2*Δ) was transformed with a single copy plasmid that expressed the Pbs2-S514D T518D mutant from its native promoter in addition to both a reporter plasmid carrying the *8xCRE-lacZ* gene and an Opy2 mutant plasmid. **(E)** Signal flow in the Hog1 MAPK cascade initiated by the constitutively-active Ste11-Q301P. Without hyperactive *OPY2* mutation, activation of Hog1 is very weak (left panel). Hyperactive *OPY2* mutation might enhance the signaling between Ste11 and Pbs2 (middle panel) or that between Pbs2 and Hog1 (right panel). Vertical arrows indicate signal flows, whereas the horizontal red arrows indicate the site of action of the hyperactive *OPY2* mutations.

In order to identify the mechanisms that might regulate the signaling efficiency of the Ste11-Pbs2-Hog1 MAPK cascade, we screened for hyperactive mutants of Opy2 that have higher function than the wild-type. For that purpose, we utilized our finding that overexpression of wild-type Opy2 (Opy2-WT) did not appreciably activate the Hog1 MAPK, even in a host cell that expressed a constitutively-active Ste11 (*e*.*g*., Ste11-Q301P) at the endogenous level [24]. Thus, we screened for Opy2 mutants that, when overexpressed, activated Hog1 in the host cell that expressed Ste11-Q301P. Activation of Hog1 was monitored by increased expression of the Hog1-dependent reporter gene *8xCRE-lacZ* (see Materials and methods for details). In this manner, we previously identified two hyperactive Opy2 mutants, Opy2-F96I and Opy2-A104V, that had a mutation in its transmembrane (TM) domain [24]. In this study, we expanded this screening by systematically mutating the Opy2 TM domain using degenerate oligonucleotides. Thus, we found two additional hyperactive mutations, Opy2-I93A and Opy2-G95L (Fig 2A). When overexpressed for 2 hours from the *GAL1* promoter, each of the three Opy2 mutants (Opy2-G95L, Opy2-F96I, and Opy2-A104V) potently activated the HOG pathway in the Ste11-Q301P cells (Fig 2B). Although the fourth mutant, Opy2-I93A, was only marginally more effective than Opy2-WT, later analyses showed that it did enhance the Opy2 function (see below).

The hyperactive Opy2 mutations are located in two separate regions in the Opy2 TM domain. I93A, G95L and F96I are close to the extracellular end of the Opy2 TM domain. Previously, using a chemical crosslinking strategy, we have shown that these three amino acid positions in Opy2 interact with Ala-124 of Sho1, which is at the extracellular end of the fourth TM domain (TM4) of Sho1 [24]. Another Opy2 hyperactive mutation, A104V, is located near the middle of the Opy2 TM domain. Because of its position deep inside the cytoplasmic membrane, it was difficult to examine if Opy2 Ala-104 interact with any Sho1 residues by the crosslinking approach. However, by co-precipitation assays, it was found that Opy2-A104V had a much higher affinity to Sho1 than Opy2-WT [24]. Thus, both sets of Opy2 hyperactive mutations seem to affect the interaction between Opy2 and Sho1.

To examine if there is any functional interaction between the two sets of Opy2 hyperactive mutations, we combined each of the three mutations in the first set (I93A, G95L and F96I) with A104V, to generate double mutants such as Opy2-I93A A104V. Overexpression of any of the four single Opy2 hyperactive mutants did not induce Hog1 activity in a cell that expressed Ste11-WT (Fig 2C). In contrast, the three double mutants substantially activated Hog1 in the same cell. Notably, I93A, which by itself had only marginal hyperactivity, synergized with A104V to a similar extent as the more strongly hyperactive G95L did. Thus, there is a strongly synergistic effect between the two sets of mutations, suggesting (though not proving) that the two sets of Opy2 hyperactive mutations enhance the Opy2 function by different mechanisms.

A known function of Opy2 is to recruit the Ste50-bound Ste11 molecule to the plasma membrane, where Ste11 interacts both with its activator Ste20 (or Cla4) and with its substrate Pbs2 [22, 33]. Thus, a potential mechanism that renders an Opy2 mutant hyperactive is to elevate its expression levels, so that the density of Ste11 on the membrane will increase. However, the expressed amounts of these four hyperactive Opy2 mutant proteins were not substantially different from that of Opy2-WT. Another potential mechanism that renders an Opy2 mutant hyperactive is to increase its affinity to the cytoplasmic adaptor protein Ste50. Indeed, we have identified Opy2 hyperactive mutants in its cytoplasmic domain that have higher affinity to Ste50 [22]. However, the same mechanism is unlikely to explain the current Opy2 mutants that are mutated in the TM domain. Thus, the hyperactive Opy2 mutations likely increased the efficiency of the Ste11-Pbs2-Hog1 MAPK cascade signaling without affecting the amount of the Ste50-Ste11 complex recruited to the plasma membrane.

### Hyperactive mutations in the Opy2 TM domain enhance activation of Pbs2 by Ste11

From the strategy of isolating the Opy2 hyperactive mutants, it is anticipated that these mutants enhance signaling along the Ste11-Pbs2-Hog1 kinase cascade. The hyperactive Opy2 mutants might enhance the signaling step from Ste11 to Pbs2 (model 1) or that from Pbs2 to Hog1 (model 2). Both models are consistent with the finding that Hog1 activation in the presence of the constitutively-active Ste11-Q301P (without osmostress) is enhanced by the hyperactive Opy2 mutants (Fig 2D, left; see Fig 2E for schematic explanation). To find out which of these two models was correct, we examined whether Hog1 activation by the constitutively-active Pbs2 S514D T518D (Pbs2-DD) [34] could be also enhanced by overexpression of the hyperactive Opy2 mutants. If the model 2 was correct, then activation of Hog1 by Pbs2-DD should be enhanced by the Opy2 hyperactive mutations. However, expression of any of the four hyperactive Opy2 mutants did not increase the activity of Hog1 any more than Opy2-WT did (Fig 2D: right), indicating that the activation of Hog1 by Pbs2 was not affected by these Opy2 hyperactive mutants. In other words, we could eliminate the model 2. Thus, it is likely that the hyperactive mutations in the Opy2 TM domain enhanced the activation/phosphorylation of Pbs2 by Ste11.

### Isolation of a hyperactive Sho1 mutant that has a mutation in one of its TM domains

While Opy2 anchors the Ste50-Ste11 complex to the plasma membrane, Sho1 anchors Pbs2 to the plasma membrane [35]. Furthermore, we have shown that Opy2 and Sho1 interact each other through their TM domains [24]. Thus, an increased affinity between Opy2 and Sho1 will be expected to enhance the interaction between Pbs2 and Ste11. In fact, we have shown previously that one of the hyperactive Opy2 mutants, Opy2-A104A, binds to Sho1 more strongly than Opy2-WT does [24], supporting the importance of the interaction between Sho1 and Opy2 in activation of the Hog1 MAPK cascade.

**Fig 3:**
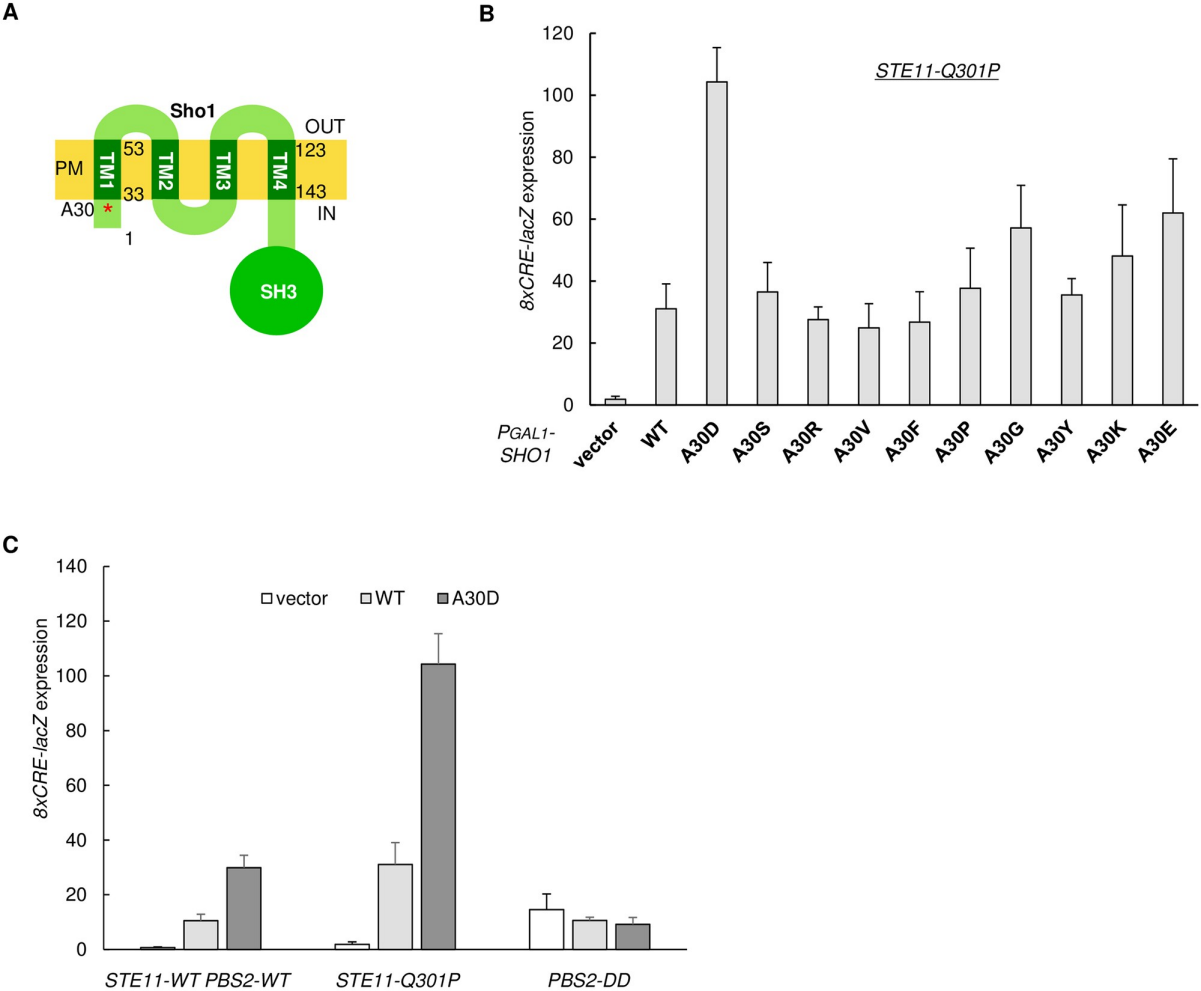
Isolation of the hyperactive Sho1-TM mutant, in which activation of Pbs2 by Ste11 is enhanced. (**A**) Diagram of the four TM domains in Sho1. Numbers indicate amino acid positions. Asterisk indicates the position of Ala-30. (**B, C**) Expression of the Hog1-specific reporter gene *8xCRE-lacZ*. β-galactosidase activity is expressed in Miller units. Error bars represent S.D. (n ≥ 3). (B) The yeast strain KT024 (*ssk2/22*Δ *sho1*Δ *STE11-Q301P*) was transformed with a reporter plasmid carrying the *8xCRE-lacZ* gene and a plasmid that expresses the indicated Sho1 mutants from the *GAL1* promoter (P_*GAL1*_). Expressions of Sho1 mutants were induced by 2% galactose for 2 hr, and expression of the *8xCRE-lacZ* gene was determined. (C) The yeast strain KT039 (*ssk2/22*Δ *sho1*Δ *pbs2*Δ) was transformed with a single copy plasmid that expressed either Pbs2-WT or Pbs2-S514D T518D mutant from its native promoter in addition to the *8xCRE-lacZ* reporter plasmid and another plasmid that expresses Sho1-WT, Sho1-A30D, or none (vector) from the *GAL1* promoter (for *PBS2-WT*, or *PBS2-DD*).

To obtain more insight into the role of the TM domain interactions between Opy2 and Sho1 in the Hog1 MAPK cascade, we isolated hyperactive mutants of Sho1 that, when overexpressed, activated Hog1 in the cells that expressed the constitutively- active Ste11-Q301P (see Materials and methods for details). Thus, we isolated a hyperactive mutant Sho1-A30D, which has an Asp substitution of Ala-30 at the intracellular end of the Sho1 TM1 domain (Fig 3A). We also created nine other substitution mutations at Sho1 Ala-30 (to S, R, V, F, P, G, Y, K, or E), but none showed any significant hyperactivity (Fig 3B).

Potentially, the *SHO1-A30D* mutation could enhance either the signaling from Ste11 to Pbs2 or that from Pbs2 to Hog1, or both. To find out which step was actually enhanced by the *SHO1-A30D* mutation, we used the same strategy as explained in Fig 2E. Thus, we examined whether activation of Hog1 by the constitutively-active Ste11-Q301P or Pbs2-DD, respectively, could be enhanced by overexpression of the hyperactive Sho1-A30D. As shown in Fig 3C, Sho1-A30D potently activated the HOG pathway (without osmostress) in the *STE11-Q301P* mutant cells, but not in the *PBS2-DD* mutant cells, indicating that the activation of Hog1 by Pbs2 was not enhanced by Sho1-A30D. Thus, it is likely that the hyperactive *SHO1-A30D* mutation, like the hyperactive Opy2 mutants shown above, enhanced the activation/phosphorylation of Pbs2 by Ste11.

### Sho1-A30D binds to Opy2 more strongly than Sho1-WT does

**Fig 4:**
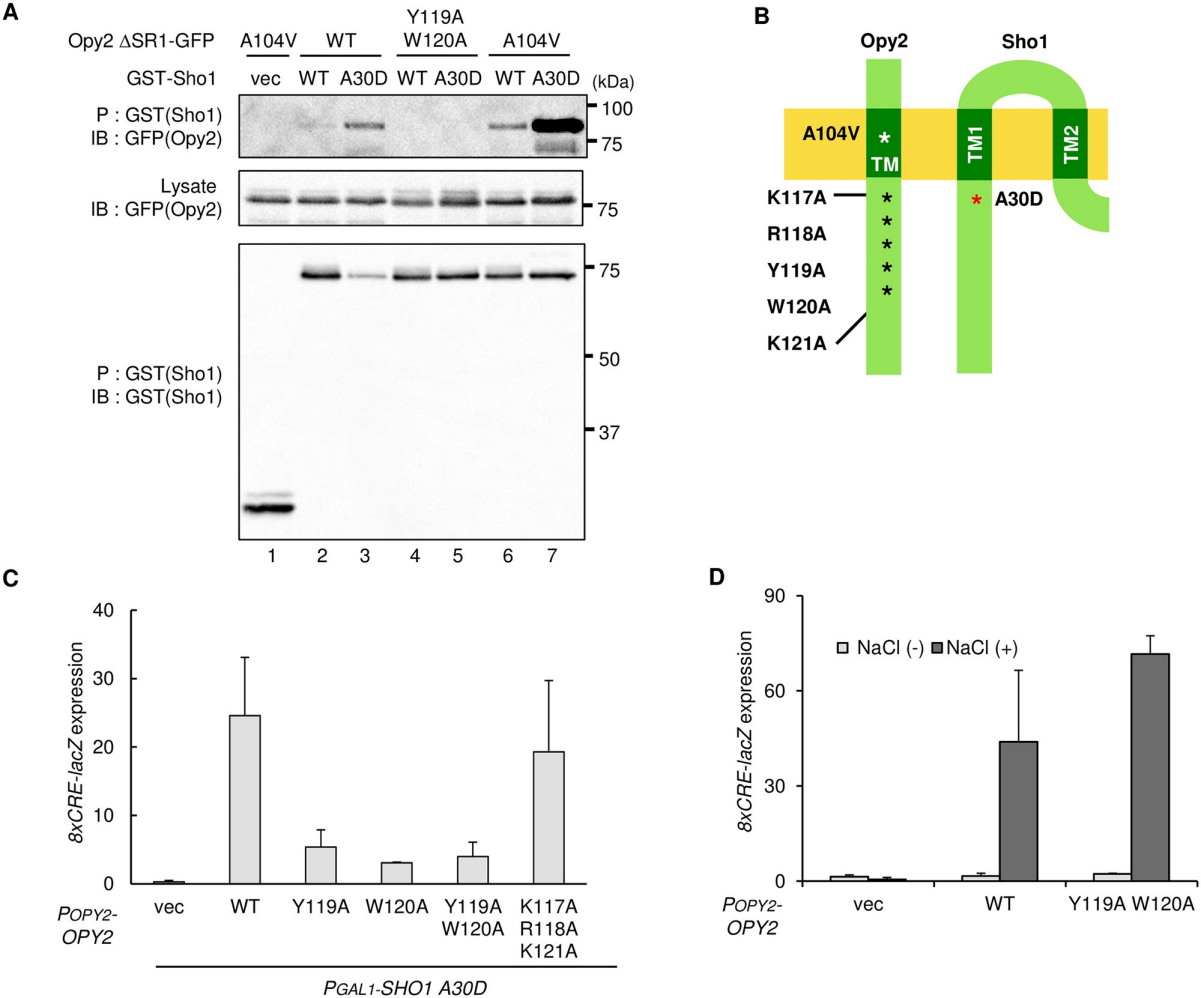
Enhanced binding of Sho1 and Opy2 by the hyperactive *SHO1-A30D* mutation. **(A**) *In vivo* co-precipitation assays between mutants of Sho1 and Opy2. The yeast strain KY590-1 (*ssk2/22*Δ *sho1*Δ *opy2*Δ) was co-transfected with the plasmids expressing the indicated derivatives of Opy2Δ SR1-GFP and GST-Sho1 (or empty vector, vec) both under the *GAL1* promoter. Transformed cells were grown in CARaf, and expression of the tagged proteins was induced by 2% galactose for 2 hr. Cell extracts were prepared using Buffer A containing 1% digitonin. GST proteins were captured by the Gluthatione -Sepharose beads (P) from cell extracts. Beads were washed three times with Buffer A containing 0.3% Brij L23, and co-precipitated proteins were detected by immunoblotting (IB) using the indicated antibodies. (**B**) Schematic model of the TM domains of Opy2 and Sho1 that interact each other. Numbers indicate amino acid positions. Asterisks indicate the positions of relevant mutations in Opy2 and Sho1. (**C, D**) Expression of the Hog1-specific reporter gene *8xCRE-lacZ*. β-galactosidase activity is expressed in Miller units. Error bars represent S.D. (n ≥ 3). (C) The yeast strain KY590-1 (*ssk2/22*Δ *sho1*Δ *opy2*Δ) was co-transformed with three plasmids: the first plasmid is a reporter plasmid carrying the *8xCRE-lacZ* gene, the second expresses the indicated Opy2 mutants from the *OPY2* native promoter, and the third expresses the Sho1-A30D mutant from the *GAL1* promoter. Expression of Sho1-A30D was induced by 2% galactose for 2 hr, and expression of the *8xCRE-lacZ* gene was determined. (D) The yeast strain KY590-1 (*ssk2/22*Δ *sho1*Δ *opy2*Δ) was co-transformed with three plasmids: one is a reporter plasmid carrying the *8xCRE-lacZ* gene, a second plasmid expresses the indicated Opy2 mutants from their native promoter, and third one expresses Sho1-WT from its native promoter. Cells were stimulated with (black) or without (white) 0.4 M NaCl for 30 min, and expression of the *8xCRE-lacZ* gene was determined.

To examine if the *SHO1-A30D* mutation affects the interaction between Opy2 and Sho1, we conducted in vivo co-precipitation experiments. An expression plasmid encoding either the N-terminally GST-tagged Sho1 (GST-Sho1), GST-Sho1-A30D, or GST alone was introduced into *sho1*Δ *opy2*Δ cells together with a second plasmid encoding the C-terminally GFP-tagged Opy2ΔSR1 (Opy2ΔSR1-GFP) or its mutant derivatives. We have deleted the extracellular Ser-rich region SR1 from all the Opy2-GFP constructs, as heterogeneous glycosylation in SR1 diffused the migration of Opy2-GFP. SR1 is not essential for the function of Opy2 in Hog1 activation [21]. The GST-Sho1 and Opy2ΔSR1-GFP proteins, whose expressions are under the control of the *GAL1* promoter, were simultaneously induced for 2 hours by addition of 2% galactose, before preparation of cell extracts. GST-Sho1 (or GST alone) was precipitated using the Glutathione Sepharose beads, and the co-precipitated Opy2ΔSR1-GFP was probed by Western blotting. GST-Sho1-WT bound to Opy2ΔSR1-GFP only weakly, but, to Opy2ΔSR1-A104V-GFP more strongly (Fig 4A lanes 2 and 6). In contrast, GST-Sho1-A30D bound to Opy2ΔSR1-GFP as strongly as GST-Sho1-WT bound to Opy2ΔSR1-A104V-GFP (Fig 4A lanes 3 and 6). Furthermore, the effects of the *SHO1-A30D* and *OPY2-A104V* mutations were additive, as the binding between Sho1-A30D and Opy2-A104V was stronger than when either partner had no mutation (Fig 4A lane 7), suggesting that the two mutations enhanced the affinity between Sho1 and Opy2 independently of each other. That the Sho1 Ala-30 residue and the Opy2 Ala-104 residue are spatially well separated (Opy2 Ala-104 is in the middle of a TM domain, whereas Sho1 A30 is at the cytoplasmic border of a TM domain; Fig 4B) is consistent with this interpretation.

### Mutations in Opy2 that suppress Hog1 activation induced by Sho1-A30D

To examine whether the enhanced binding between Sho1 and Opy2 is directly responsible for the increased Hog1 activation by Sho1-A30D, we screened for *OPY2* mutants that suppressed the Hog1 activation induced by expression of Sho1-A30D. Because the Sho1 Ala-30 residue resides at the cytoplasmic border of the Sho1 TM1 domain, we focused on the Opy2 residues also at the cytoplasmic border of its TM domain. In brief, we constructed by site-directed mutagenesis Opy2 mutants in which residues 117–121 were either singly or multiply replaced by Ala (Fig 4B). We initially thought it likely that the three positively charged residues in Opy2, namely K117, R118, and K121, were responsible for its increased affinity to Sho1-A30D by enhanced electrostatic attractions. However, substitution of K117, R118, and K121 with Ala did not affect the activation of Hog1 induced by Sho1-A30D (Fig 4C). In contrast, replacements of the aromatic residues Y119 and/or W120 with Ala drastically suppressed Hog1 activation induced by Sho1-A30D. If these *OPY2* mutations had inactivated a function of Opy2 that was necessary for Hog1 activation, then they would have also inhibited the osmostress-induced Hog1 activation. However, the Opy2-Y119A W120A double mutant supported osmostress-induced Hog1 activation to a similar extent as Opy2 WT did (Fig 4D). Thus, the Opy2-Y119A W120A mutant protein retains the normal functions of Opy2, but it specifically suppresses the signaling enhancing effect of the *SHO1-A30D* mutation.

We then investigated whether or not the *OPY2-Y119A W120A* mutation affected the binding between Sho1 and Opy2. In vivo co-precipitation experiments showed that introduction of the *OPY2-Y119A W120A* mutation completely abrogated the enhanced binding between Sho1-A30D and Opy2-WT (Fig 4A, lanes 3 and 5). Thus, the enhanced Hog1 activation by Sho1-A30D is due to its higher affinity to Opy2.

**Fig 5:**
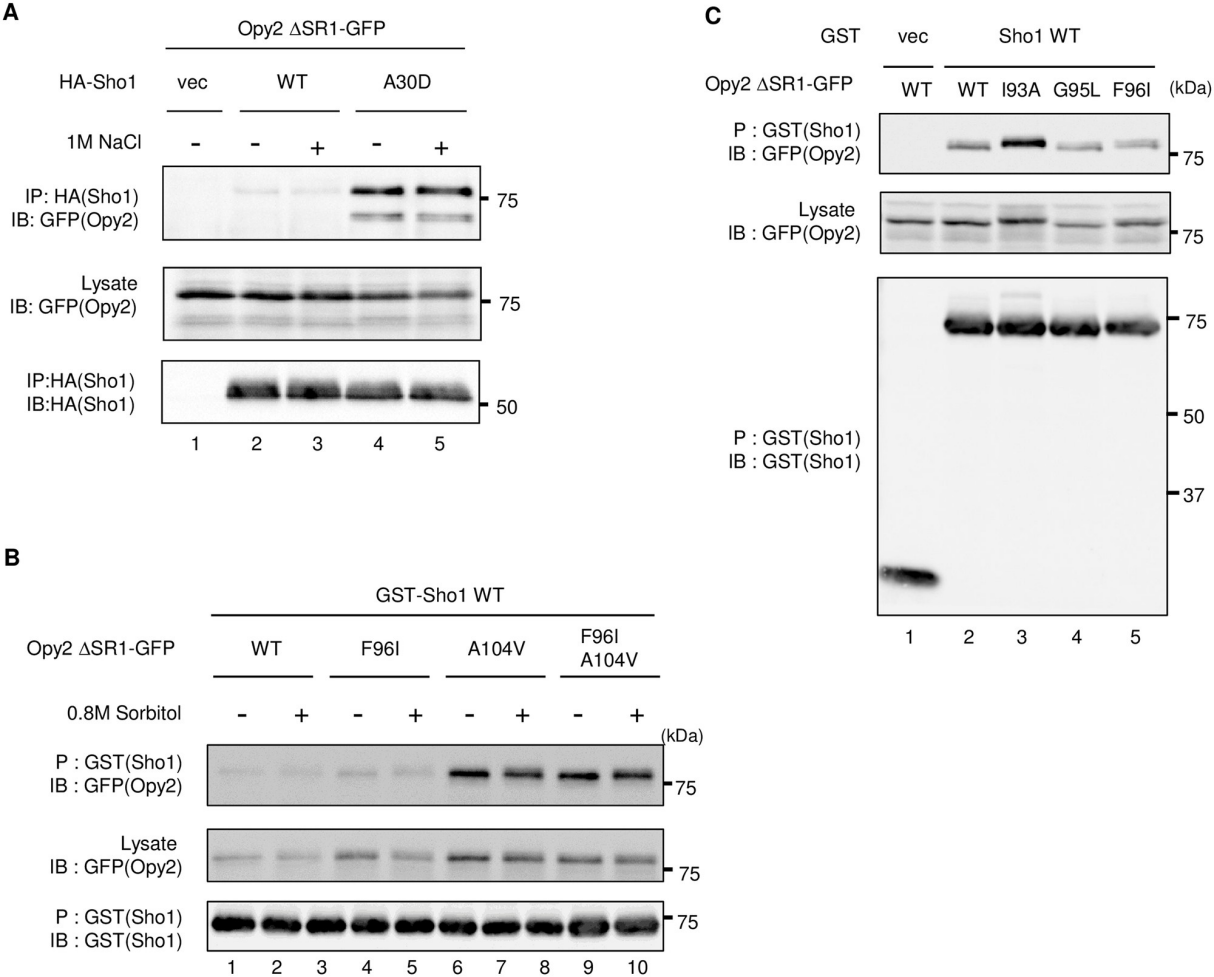
Effects of the mutations and osmotic stresses on the *in vivo* binding between Sho1 and Opy2. (**A**-**C**) *In vivo* binding between Sho1 and Opy2 was assayed by co-precipitation of Sho1 and Opy2. The yeast strain TM257 (*ssk2/22*Δ) (A), FP75 (*ssk2/22*Δ *ste11*Δ) (B), or KY590-1(*ssk2*Δ *ssk22*Δ *sho1*Δ *opy2*Δ) (C) was co-transfected with a plasmid expresses the indicated derivatives of Opy2Δ SR1-GFP and another plasmid that expresses either GST-Sho1 (A and B) or HA-Sho1 (C) all under the *GAL1* promoter. vec, empty vector. Transformed cells were grown in CARaf, and expression of the tagged proteins was induced by 2% galactose for 2 hr. Sorbitol (0.8M, final conc.) was added (+) for 3 min (B), or not added (-). Cell extracts were prepared using Buffer A containing the following detergents: (A) 0.3% Brij-L23, (B) 0.1% Brij-97, (C) 1% digitonin. (A) HA-Sho1 was immunoprecipitated (IP) from cell extracts using anti-HA antibody and Protein G Sepharose beads, whereas (B and C) GST proteins were captured by the Gluthatione-Sepharose beads. In all cases, beads were washed three times with the same buffer used for cell lysate preparation, and co-precipitated proteins were detected by immunoblotting (IB) using the indicated antibodies.

We next considered the possibility that the interaction between Opy2 and Sho1 is induced, or enhanced, by the external osmostress. Our co-precipitation experiments, however, indicated that the binding between Opy2 and Sho1 was not influenced by high osmolarity (Fig 5A and 5B). Thus, although the increased affinity between Sho1 and Opy2 results in enhanced activation of Pbs2 by Ste11, the affinity between Sho1 and Opy2 is not directly influenced by the external osmostress. Interestingly, some hyperactive Opy2 mutants (such as Opy2-G95L and Opy2-F96I), increased Hog1 signaling without increasing the Opy2-Sho1 binding (Fig 5C). Thus, there seems a mechanism that enhances activation of Pbs2 by Ste11 without changing the affinity between Sho1 and Opy2.

### Close interaction between the Opy2 TM domain and the Sho1 TM1 domain

Previously, using a chemical crosslinking strategy, we have shown that the extracellular end of the Opy2 TM domain interacts with the extracellular end of the Sho1 TM4 domain [24]. However, little was known about how the Opy2 and Sho1 TM domains interact at their cytoplasmic ends. In this study, we have shown that the *OPY2-Y119A W120A* mutation near the cytoplasmic end of the Opy2 TM domain suppressed the *SHO1-A30D* mutation near the cytoplasmic end of the Sho1 TM1 domain. This finding suggested that the cytoplasmic end of the Opy2 TM domain interacted with the cytoplasmic end of the Sho1 TM1 domain. This possibility was examined using a site-directed cysteine (Cys) chemical crosslinking strategy developed by Wu and Kaback [36], which had been adapted previously by us to analyze the Sho1 multimerization [24].

**Fig 6:**
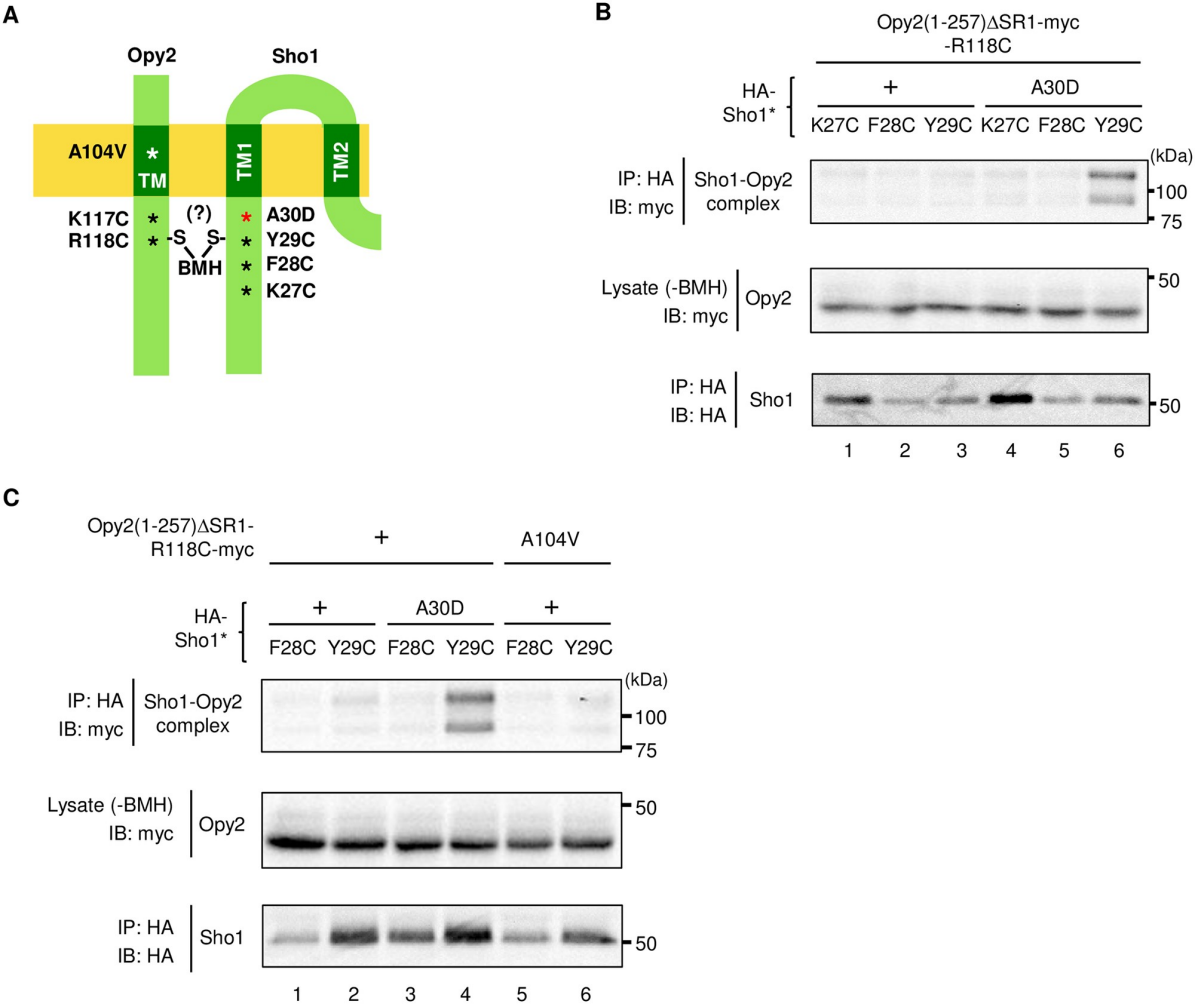
Close interaction between the Opy2 TM domain and the TM1 domain of Sho1. (**A**) Schematic model of the TM domains of Opy2 and Sho1 that interact each other. Numbers indicate amino acid positions. Asterisks indicate the positions of relevant mutations in Opy2 and Sho1. (**B, C**) Chemical crosslinking between Sho1 and Opy2. Expression plasmids (*GAL1* promoter) for the indicated mutant derivatives of HA-Sho1* and either Opy2(1–257) ΔSR1-myc-R118C or A104V R118C were co-transfected into KY590-1. After induction by 2% galactose for 2 h, intact cells were treated with 0.4 mM BMH at 30 °C for 5 min. For the control samples to monitor the Opy2-myc expression levels, BMH treatment was omitted (-BMH)). Cell lysates were prepared with buffer A containing 0.2% Triton X-100. HA-Sho1* was immunoprecipitated, and the covalently bound Sho1-Opy2 complex was detected by immunoblotting.

Initially, we generated Cys-substitution mutants of the Opy2 TM domain and the Sho1 TM1 domain at the residues near their cytoplasmic boundaries (Fig 6A). To construct the Sho1 Cys-substitution mutants for crosslinking experiments, we used the N-terminally HA-tagged Sho1* (HA-Sho1*) and its A30D derivative (HA-Sho1*-A30D). Sho1* is a Cys-free derivative of Sho1 in which the two native Cys residues were substituted with Ser [24]. As for Opy2, Cys mutation was introduced into the myc-tagged Opy2(1–257) ΔSR1. One of the Sho1 Cys substitution mutants was co-expressed with one of the Opy2 Cys substitution mutants in an *sho1*Δ *opy2*Δ strain, and genes were induced from the *GAL* promoter for 2 hr in the presence of 2% galactose. Then, the Cys-substitution mutant proteins were crosslinked with 1,6-bis-maleimidohexane (BMH) *in vivo* for 5 min, as previously described [24]. When a Cys residue in Sho1 and another Cys residue in Opy2 are close together (6-16Å), they can be crosslinked by BMH to form a covalent bound Sho1-Opy2 heterodimer. The cell lysates were prepared using a Triton X-100 containing buffer to disrupt the non-covalent binding between Sho1 and Opy2 [24]. HA-Sho1 was immunoprecipitated, and the covalently-bound HA-Sho1/Opy2-myc heterodimer was probed by anti-myc antibody.

The Cys residue at the position R118 in Opy2 (namely, Opy2 R118C) was efficiently crosslinked to Y29C of Sho1, but only in the presence of *SHO1-A30D* mutation (Fig 6B, lanes 3 and 6). Opy2 R118C was crosslinked neither to Sho1 K27C nor Sho1 F28C (lanes 1, 2, 4 and 5). That the crosslinking between Opy2 R118C and Sho1 Y29C was observed only in the presence of the *SHO1-A30D* mutation might merely be due to the higher affinity of Opy2 to Sho1-A30D than to Sho1-WT. If so, another mutation that increases the affinity between Opy2 and Sho1, namely *OPY2-A104V*, should also enhance the crosslinking between Opy2 R118C and Sho1 Y29C. As we have shown in Fig 4A, the *OPY2-A104V* mutation increases the affinity between Opy2 and Sho1 to a similar extent as the *SHO1-A30D* does. However, *OPY2-A104V* did not enhance the crosslinking between Opy2 and Sho1 (Fig 6C, compare lanes 4 and 6). Thus, the *SHO1-A30D* mutation, in addition to increasing the affinity between Opy2 and Sho1, must somehow increases the probability of crosslinking Opy2 R118C and Sho1 Y29C in an Opy2-Sho1 complex, perhaps by altering the local conformation of Sho1 Y29C. Taken together, it is likely that, in the Opy2-Sho1 complex, the cytoplasmic end of the Opy2 TM domain is physically closely associated with the cytoplasmic end of the Sho1 TM1 domain.

## Discussion

In the SHO1 branch of the HOG pathway, the four-tiered protein kinase cascade Ste20 (or Cla4)-Ste11-Pbs2-Hog1 transduces the signal for environmental osmotic stress to the nucleus. Specific and efficient signal flow through this cascade is essential for prompt adaptation to osmostress, and is realized by multiple mechanisms. First, the consecutive kinases in the cascade bind to each other through the specific docking mechanisms [26, 31]. Second, the kinases in the first three tiers are all localized to the plasma membrane, by binding to their respective membrane-anchoring proteins: Ste20/Cla4 by the membrane-associated Cdc42 [37, 38]; Ste11 by the Opy2-Ste50 complex [22, 33]; and Pbs2 by the Sho1 membrane protein [18, 35, 39]. When they are localized on the plasma membrane, these kinases can encounter each other more efficiently than when they are in the cytoplasm. Hog1 itself, however, is not membrane-localized, perhaps because it must rapidly shuttle between the cytoplasm and the nucleus. Third, the membrane anchor proteins Opy2 and Sho1 bind to each other, thus further increasing the efficiency of interaction between Ste11 and Pbs2.

Isolation of hyperactive mutants often shed light on the activation mechanisms of the signaling pathway in which the mutated proteins are rate-limiting. In this work, we isolated hyperactive mutants of Opy2 and Sho1 that increased the efficiency of Hog1 activation. The isolated Opy2 and Sho1 mutants all had mutations in their TM domains. Some mutants, such as Opy2-I93A, Opy2-A104V and Sho1-A30D, enhanced the binding strength between Opy2 and Sho1. The increased association between Opy2 and Sho1 will increases the interaction between Ste11 and Pbs2, thus enhancing the signaling between them. Indeed, our genetic analyses indicated that the mutants that increased interaction between Opy2 and Sho1 enhanced the activation of Pbs2 by Ste11, but not that of Hog1 by Pbs2. Thus, we initially considered the possibility that an induced association between Opy2 and Sho1 might play a role in the osmotic activation of Pbs2 by Ste11. Our co-precipitation experiments, however, indicated that the binding between Opy2 and Sho1 was not influenced by high osmolarity. Interestingly, some other hyperactive Opy2 mutants (such as Opy2-G95L and Opy2-F96I), increased the efficiency of the Hog1 signaling without increasing the Opy2-Sho1 binding. This finding suggested to us another possibility that the signaling efficiency between Ste11 and Pbs2 might be modulated by a conformational change in the Opy2-Sho1 complex. Consistent with this idea, we found that Opy2 and Sho1 interacted at two distinct binding sites. We have described previously that in the Opy2-Sho1 complex the Opy2 residues Gly-95/Phe-96 are closely associated with the Sho1 residue Ala-124 [24]. These residues thus form the binding site 1 (BS-1) between the Opy2 TM domain and the Sho1 TM domain 4 (TM4) at their respective extracellular ends. In this work, we revealed that Sho1-A30D, a mutation at the cytoplasmic end of the Sho1 TM domain 1 (TM1), enhanced Sho1-Opy2 binding. Furthermore, using chemical crosslinking strategy, we showed that the Opy2 residue Arg-118 (at the cytoplasmic end of the Opy2 TM domain) is closely associated with the Sho1 residue Tyr-29 (at the cytoplasmic end of the Sho1 TM1). These residues thus are likely to form the second binding site (BS-2) between the Opy2 TM domain and the Sho1 TM1 domain.

**Fig 7:**
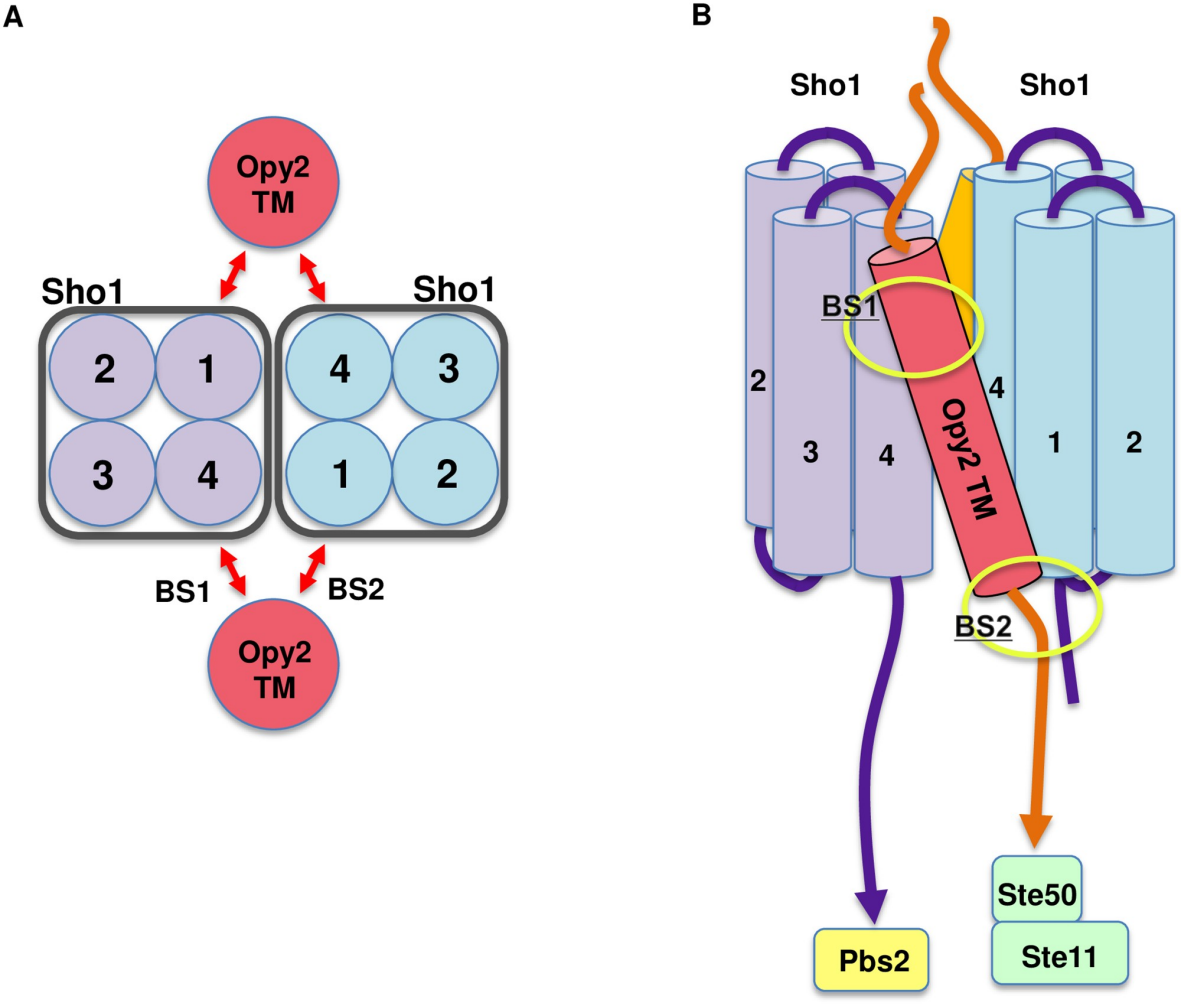
Schematic model of the transmembrane interactions between Sho1 and Opy2. **(A)** A top view of the interaction between an Sho1 dimer and two Opy2 monomers. Light purple/blue circles with numbers represent the four TM domains of Sho1. Red circles represent the TM domain of Opy2. Red arrows indicate the TM-TM interactions between Sho1 and Opy2 at the binding sites 1 and 2 (BS1 and BS2). **(B)** A side view of the interactions among an Sho1 dimer and two Opy2 monomers. Transmembrane (TM) domains of Sho1 and Opy2 are indicated as light purple/blue and red cylinders, respectively. Extracellular and cytoplasmic regions that are connected with TM domains are partly shown as a line. BS1 and BS2 are indicated by yellow ovals.

To speculate on the potential significance of the presence of two binding sites between Opy2 and Sho1, it is necessary first to review the oligomeric structure of Sho1. Sho1, with its four tightly packed TM domains (TM1—TM4), forms two separate interfaces for binding to other Sho1 molecules [24]. In the Sho1 molecule, its TM1 and TM4 domains are juxtaposed to constitute an interface through which two Sho1 molecules bind to form a dimeric subassembly. Furthermore, TM2 and TM3 of a Sho1 molecule constitute the second interface through which three Sho1 molecules form a trimeric subassembly. Sho1 oligomers of undefined sizes are generated by alternative employments of the two types of the Sho1-Sho1 interfaces. Because Sho1 dimerizes at the TM1/TM4 interface, it will be difficult for Opy2 to interact with both the TM1 and TM4 domains of the same Sho1 molecule. It is more plausible that Opy2 interacts with two Sho1 molecules, one at the cytoplasmic end of TM1 and another at the extracellular end of TM4 (Fig 7A). Thus, Ste11, which is bound to the Opy2 intracellular domain via Ste50, will be placed directly below the Sho1 TM1 domain. On the other hand, Pbs2, which is bound to the cytoplasmic SH3 domain of Sho1, is placed below the Sho1 TM4 domain (Fig 7B). This structural arrangement might be important to place Ste11 and Pbs2 in a close proximity for efficient signaling.

It seems possible that the Sho1-Opy2 complex dynamically controls the positional relationship, and thus signaling efficiency, between Ste11 and Pbs2, by a kind of leverage mechanism. Because the extracellular end and the cytoplasmic end of the Opy2 TM domain are fixed to different molecules of Sho1, the tilt angle (relative to the membrane plane) of the Opy2 TM domain might be very sensitive to the structural distortion of the Sho1 oligomers that might be induced by osmotic stress. A small change in the Opy2 tilt angle will then be amplified by the leverage mechanism to a significant spatial relationship between Ste11 and Pbs2. This might be a mechanism by which the osmosensor Sho1 controls Hog1 activation. Properties of Opy2 and Sho1 hyperactive mutants reported here and previously are consistent with this model. Several hyperactive mutants within the Opy2-Sho1 binding site 1 (Opy2-G95L and F96I) did not enhance the binding strength between Opy2 and Sho1 (Fig 5C). These mutations might mimic the osmostress conditions by changing the structural relationship between Opy2 and Sho1. Other hyperactive mutants of Sho1 have been found in the extracellular loop between TM3 and TM4 (for examples, Sho1-P120L and P120V) [19]. So far, there is no explanation for the hyperactivity of these mutants, but they might also mimic the conformational changes in the Sho1 oligomer induced by osmostress.

A crucial assumption in the above hypothesis is that an external osmostress induces a structural change in the Sho1 oligomer. A 2-dimensioanl lattice of TM domains in Sho1 oligomer seems an ideal device to detect small distortions of the plasma membrane caused by osmostress. In fact, we have found that osmostress induces structural change in the Sho1 TM domains, using chemical crosslinking analyses of Sho1 mutants that contain a cysteine substitution mutation at various positions in its TM domains [24]. Detailed structural analyses of both the Sho1 oligomer and the Opy2-Sho1 complex will be highly rewarding to understand the dynamic activation mechanism of the Hog1 MAPK by osmostress.

## References

[1] Albertyn J, Hohmann S, Thevelein JM, Prior BA. *GPD1*, which encodes glycerol-3-phosphate dehydrogenase, is essential for growth under osmotic stress in *Saccharomyces cerevisiae*, and its expression is regulated by the high-osmolarity glycerol response pathway. Mol Cell Biol. 1994;14:4135–44. 8196651 10.1128/mcb.14.6.4135-4144.1994 PMC358779

[2] Blomberg A, Adler L. Roles of glycerol and glycerol-3-phosphate dehydrogenase (NAD^+^) in acquired osmotolerance of *Saccharomyces cerevisiae*. J Bacteriol. 1989;171(2):1087–92. 2644223 10.1128/jb.171.2.1087-1092.1989 PMC209705

[3] Brewster JL, de Valoir T, Dwyer ND, Winter E, Gustin MC. An osmosensing signal transduction pathway in yeast. Science. 1993;259:1760–3. 7681220 10.1126/science.7681220

[4] Lee J, Reiter W, Dohnal I, Gregori C, Beese-Sims S, Kuchler K, et al MAPK Hog1 closes the *S*. *cerevisiae* glycerol channel Fps1 by phosphorylating and displacing its positive regulators. Genes Dev. 2013;27:2590–601. 10.1101/gad.229310.113 24298058 PMC3861672

[5] Ferreira C, van Voorst F, Martins A, Neves L, Oliveira R, Kielland-Brandt MC, et al A member of the sugar transporter family, Stl1p is the glycerol/H^+^ symporter in *Saccharomyces cerevisiae*. Mol Biol Cell. 2005;16(4):2068–76. 10.1091/mbc.E04-10-0884 15703210 PMC1073684

[6] O'Rourke SM, Herskowitz I. Unique and redundant roles for HOG MAPK pathway components as revealed by whole-genome expression analysis. Mol Biol Cell. 2004;15:532–42. 10.1091/mbc.E03-07-0521 14595107 PMC329229

[7] Warringer J, Hult M, Regot S, Posas F, Sunnerhagen P. The HOG pathway dictates the short-term translational response after hyperosmotic shock. Mol Biol Cell. 2010;21(17):3080–92. 10.1091/mbc.E10-01-0006 20587780 PMC2930000

[8] de Nadal E, Posas, F. Regulation of Gene Expression in Response to Osmostress by the Yeast Stress-Activated Protein Kinase Hog1 In: Eds S, editor. Stress-Activated Protein Kinases Topics in Current Genetics. Stress-Activated Protein Kinases. Topics in Current Genetics. 20. Heidelberg 2008 p. 81–97.

[9] Alexander MR, Tyers M, Perret M, Craig BM, Fang KS, Gustin MC. Regulation of cell cycle progression by Swe1p and Hog1p following hypertonic stress. Mol Biol Cell. 2001;12(1):53–62. 10.1091/mbc.12.1.53 11160822 PMC30567

[10] Clotet J, Escoté X, Adrover MA, Yaakov G, Garí E, Aldea M, et al Phosphorylation of Hsl1 by Hog1 leads to a G2 arrest essential for cell survival at high osmolarity. EMBO J. 2006;25(11):2338–46. 10.1038/sj.emboj.7601095 16688223 PMC1478172

[11] Adrover M, Zi Z, Duch A, Schaber J, González-Novo A, Jimenez J, et al Time-dependent quantitative multicomponent control of the G₁-S network by the stress-activated protein kinase Hog1 upon osmostress. Sci Signal. 2011;4(192):ra63 10.1126/scisignal.2002204 21954289

[12] Saito H, Posas F. Response to hyperosmotic stress. Genetics. 2012;192:289–318. 10.1534/genetics.112.140863 23028184 PMC3454867

[13] Hohmann S. Control of high osmolarity signalling in the yeast *Saccharomyces cerevisiae*. FEBS Lett. 2009;583:4025–9. 10.1016/j.febslet.2009.10.069 19878680

[14] Hohmann S. Osmotic stress signaling and osmoadaptation in yeasts. Microbiol Mol Biol Rev. 2002;66:300–72. 10.1128/MMBR.66.2.300-372.2002 12040128 PMC120784

[15] Chen Z, Gibson TB, Robinson F, Silvestro L, Pearson G, Xu B, et al MAP kinases. Chem Rev. 2001;101:2449–76. 11749383 10.1021/cr000241p

[16] van Drogen F, O'Rourke SM, Stucke VM, Jaquenoud M, Neiman AM, Peter M. Phosphorylation of the MEKK Ste11p by the PAK-like kinase Ste20p is required for MAP kinase signaling *in vivo*. Curr Biol. 2000;10:630–9. 10837245 10.1016/s0960-9822(00)00511-x

[17] Tatebayashi K, Yamamoto K, Tanaka K, Tomida T, Maruoka T, Kasukawa E, et al Adaptor functions of Cdc42, Ste50, and Sho1 in the yeast osmoregulatory HOG MAPK pathway. EMBO J. 2006;25:3033–44. 10.1038/sj.emboj.7601192 16778768 PMC1500976

[18] Raitt DC, Posas F, Saito H. Yeast Cdc42 GTPase and Ste20 PAK-like kinase regulate Sho1-dependent activation of the Hog1 MAPK pathway. EMBO J. 2000;19:4623–31. 10.1093/emboj/19.17.4623 10970855 PMC302074

[19] Tatebayashi K, Tanaka K, Yang H-Y, Yamamoto K, Matsushita Y, Tomida T, et al Transmembrane mucins Hkr1 and Msb2 are putative osmosensors in the SHO1 branch of yeast HOG pathway. EMBO J. 2007;26:3521–33. 10.1038/sj.emboj.7601796 17627274 PMC1949007

[20] Tanaka K, Tatebayashi K, Nishimura A, Yamamoto K, Yang H-Y, Saito H. Yeast osmosensors Hkr1 and Msb2 activate the Hog1 MAPK cascade by different mechanisms. Science Signaling. 2014;7:ra21 10.1126/scisignal.2004780 24570489

[21] Yang H-Y, Tatebayashi K, Yamamoto K, Saito H. Glycosylation defects activate filamentous growth Kss1 MAPK and inhibit osmoregulatory Hog1 MAPK. EMBO J. 2009;28:1380–91. 10.1038/emboj.2009.104 19369942 PMC2688530

[22] Yamamoto K, Tatebayashi K, Tanaka K, Saito H. Dynamic cotrol of yeast MAP kinase network by induced association and dissociation between the Ste50 scaffold and the Opy2 membrane anchor. Mol Cell. 2010;40:87–98. 10.1016/j.molcel.2010.09.011 20932477

[23] Nishimura A, Yamamoto K, Oyama M, Kozuka-Hata H, Saito H, Tatebayashi K. Scaffold protein Ahk1, which associates with Hkr1, Sho1, Ste11, and Pbs2, inhibits cross talk signaling from the Hkr1 osmosensor to the Kss1 mitogen-activated protein kinase. Mol Cell Biol. 2016;36:1109–23. 10.1128/MCB.01017-15 26787842 PMC4800789

[24] Tatebayashi K, Yamamoto K, Nagoya M, Takayama T, Nishimura A, Sakurai M, et al Osmosensing and scaffolding functions of the oligomeric four-transmembrane domain osmosensor Sho1. Nat commun. 2015;6:6975 10.1038/ncomms7975 25898136 PMC4411306

[25] Rose MD, Broach JR. Cloning genes by complementation in yeast. Methods Enzymol. 1991;194:195–230. 2005788 10.1016/0076-6879(91)94017-7

[26] Tatebayashi K, Takekawa M, Saito H. A docking site determining specificity of Pbs2 MAPKK for Ssk2/Ssk22 MAPKKKs in the yeast HOG pathway. EMBO J. 2003;22:3624–34. 10.1093/emboj/cdg353 12853477 PMC165623

[27] Posas F, Witten EA, Saito H. Requirement of STE50 for osmostress-induced activation of the STE11 mitogen-activated protein kinase kinase kinase in the high-osmolarity glycerol response pathway. Mol Cell Biol. 1998;18:5788–96. 9742096 10.1128/mcb.18.10.5788 PMC109165

[28] Sikorski RS, Hieter P. A system of shuttle vectors and yeast host strains designed for efficient manipulation of DNA in *Saccharomyces cerevisiae*. Genetics. 1989;122:19–27. 2659436 10.1093/genetics/122.1.19 PMC1203683

[29] Foreman PK D R. Cloning vectors for the synthesis of epitope-tagged, truncated and chimeric proteins in Saccharomyces cerevisiae. Gene. 1994;144(1):63–8. 7517907 10.1016/0378-1119(94)90204-6

[30] Mumberg D, Müller R, Funk M. Regulatable promoters of *Saccharomyces cerevisiae*: comparison of transcriptional activity and their use for heterologous expression. Nucl Acids Res. 1994;22:5767–8. 7838736 10.1093/nar/22.25.5767 PMC310147

[31] Murakami Y, Tatebayashi K, Saito H. Two adjacent docking sites in the yeast Hog1 Mitogen-activated protein (MAP) kinase differentially interact with the Pbs2 MAP kinase kinase and the Ptp2 protein tyrosine phosphatase. Mol Cell Biol. 2008;28:2481–94. 10.1128/MCB.01817-07 18212044 PMC2268422

[32] Horie T, Tatebayashi K, Yamada R, Saito H. Phosphorylated Ssk1 prevents unphosphorylated Ssk1 from activating the Ssk2 MAP kinase kinase kinase in the yeast HOG osmoregulatory pathway. Mol Cell Biol. 2008;28:5172–83. 10.1128/MCB.00589-08 18573873 PMC2519728

[33] Wu C, Jansen G, Zhang J, Thomas DY, Whiteway M. Adaptor protein Ste50p links the Ste11p MEKK to the HOG pathway through plasma membrane association. Genes Dev. 2006;20:734–46. 10.1101/gad.1375706 16543225 PMC1413288

[34] Wurgler-Murphy SM, Maeda T, Witten EA, Saito H. Regulation of the *Saccharomyces cerevisiae HOG1* mitogen-activated protein kinase by the *PTP2* and *PTP3* protein tyrosine phosphatases. Mol Cell Biol. 1997;17:1289–97. 9032256 10.1128/mcb.17.3.1289 PMC231854

[35] Maeda T, Takekawa M, Saito H. Activation of yeast PBS2 MAPKK by MAPKKKs or by binding of an SH3-containing osmosensor. Science. 1995;269:554–8. 7624781 10.1126/science.7624781

[36] Wu J, Kaback HR. Helix proximity and ligand-induced conformational changes in the lactose permease of *Escherichia coli* determined by site-directed chemical crosslinking. J Mol Biol. 1997;270:285–93. 10.1006/jmbi.1997.1099 9236129

[37] Peter M, Neiman AM, Park H-O, van Lohuizen M, Herskowitz I. Functional analysis of the interaction between the small GTP binding protein Cdc42 and the Ste20 protein kinase in yeast. EMBO J. 1996;15:7046–59. 9003780 PMC452530

[38] Cvrcková F, De Virgilio C, Manser E, Pringle JR, Nasmyth K. Ste20-like protein kinases are required for normal localization of cell growth and for cytokinesis in budding yeast. Genes Dev. 1995;9:1817–30. 7649470 10.1101/gad.9.15.1817

[39] Reiser V, Salah SM, Ammerer G. Polarized localization of yeast Pbs2 depends on osmostress, the membrane protein Sho1 and Cdc42. Nat Cell Biol. 2000;2:620–7. 10.1038/35023568 10980703

